# Dental Therapeutics

**Published:** 1871-10

**Authors:** F. Y. Clark

**Affiliations:** Savannah, Geo.


### ARTICLE II.

### Dental Therapeutics.

BY F. Y. CLARK, M. D., D. D. S., SAVANNAH, GEO.

The cause of caries of the teeth as far back as we can go has been a mooted question. It is called Decay, Gangrene, Mortification, Inflammation, &c., and is attributed by various authors to as many causes.

In this report we wish to call your attention to what we will designate external and internal caries. The one is hereditary and constitutional, and caused by hereditary or constitutional disease. The other is external and local, and caused by external or local agents.

Had man never violated the natural and fixed laws of God, we believe he would never have been afflicted with disease. No natural law can be violated without a penalty. Thus it has been from the entrance of our fore-parents into the garden of Eden, and will be to the end of time.

If any one member of our body suffers from disease all other members and parts sympathize. The teeth are so organized that they depend on the general system for nourishment, health, and life. Although different in structure, and more isolated than the hands and feet, they are supplied with the same kind of nerves, veins and arteries, and these little aqueducts convey the supply as given to them at the fountain head. It will be seen then that if this system is diseased that these aqueducts must carry these diseased fluids to the teeth and that they must necessarily partake of the malady. Thus we frequently see, and are called on to treat scrofulitic, syphilitic, consumptive, and dyspeptic teeth, for these and many other diseases are as well known by the well informed dentist, and as early detected on the teeth, as they are by the physician in the system. Many of these diseases, have remote origin, and it is thus "the sins of the parents are visited upon their children even to the third and fourth generation." Form this it will be readily seen that it is sometimes no easy matter to answer the oft put question, " why is it my teeth decay, I am continually having them fixed yet they are giving away ?" In many cases we might answer, "fixing without constitutional treatment will not save your teeth," and to go a little farther if we wished to get at the cause we would have to pry into family secrets, and find the vice commencing with some great grand-parent.

In hereditary disease of the teeth of this nature the best that the dentist can do is to act in concert with the family physician, for it is to him that the treatment more especially belongs. Perhaps if there was some power to regulate the marriage rite, and those competent to judge, say who should and who should not marry, then there might be some hope of freeing our race of these diseases, but as this is out of the question our treatment cannot be in many cases more than paliative. If the mouth and teeth are kept in a good con dition by the dentist, the physician's treatment will prove much more successful. In the use of acids in the treatment of nervous, hereditary, or constitutional complaints physicians are too careless, for many preparations used combine with the lime or fibre of the teeth and do irreparable mischief. In the use of such agents it is always best to act in concert with the dentist, for he knowing the nature of the teeth and what agents will have an injurious effect upon them can better advise the manner of administering such medicines. As a general rule, acids should be taken through a glass tube, straw, or quill, and the mouth rinsed with an alkaline solution immediately after.

Another cause of caries or defective teeth arises from the unnatural habits and life of mothers and nurses. How is it possible for a mother to bear a healthy offspring when the shoes on her feet oblige her to stand and walk in a stooped position known as the Grecian bend. This with tight ligatures about the waist and other parts of the body, impeding circulation, and stimulating instead of nutritious diet does much towards imperfect dentition and faulty structure of the teeth, for it is well known that whatever is wrong with the mother injures the child. Again the nurse uses the most stimulating food and drink and if the child partakes of anything besides the milk thus poisoned it is of the same nature. Is it then any wonder that these bowel complaints should be so common and fatal among our American children? We think if physicians would try and make their patients understand this better, that Bateman's drops, Paragoric, and the various Soothing Syrups would not be in such demand, and mortality among our children during the period of teething would not be so great.

Again, if the existing code of raising children is carried out; if fashionable dress is substitued for the free and easy robe; if wheeled about the streets in fashionable hand carriages with their little hands and feet pinioned as if in stocks instead of the natural out-door rough and tumble romp; if the natural taste is bribed and an artificial one is made to take its place; if highly seasoned food and acids are used along with hot and cold fluids above and below the temperature of the blood; if this along with the usual drugging is carried out then we have a state of things little intended by nature and generally beyond our skill, "for nature once resisted in his course breeds frightful things."

Obstructed dentition, owing partially to the untimely loss of the deciduous teeth. The undue erupting of a tooth here and there with no adjacent members to confine it within due limit will ere long create irregularity, a contracted arch, and early decay. It is generally about this time that the fond parent brings the child to the dentist believing that everything can be righted in a few minutes and when told that the four molars are permanent teeth and beyond saving, and that the correcting of the irregularity and filling other teeth will involve weeks and fifty or a hundred dollars, they are astonished and exclaim, they had no idea these molars were permanent or that the teeth ineeded anything that would take more than a few minutes

When the teeth of children are wanting in bone structure, phosphate of lime and other prescriptions have been by many prescribed. We have little faith in these things as a medium, but as a food with the mother and child unlimited confidence.

We might, if we would, learn many good lessons from the animal creation in this respect. They never fail to rear their young because their bodies are not distorted by fashion, and they in their natural state live on the food intended. Their instincts in many ways serves them better than our reason serves us.

Before dismissing this part of our report we would request the consideration of the profession to the great importance of saving deciduous teeth until nature gives indication that their work has been accomplished. We feel that too little attention has been paid to this subject. As a general rule we consider the retention of these teeth until the allotted time, necessary to the securing of good order and regularity in their successors. We would therefore, strongly recommend their filling whenever decayed. As the object is to retain these teeth but for a short time, we think amalgam or oxychloride zinc will answer better than any other material, for in many cases it would be impossible to use anything else. If amalgam is preferred we would recommend that of Dr. Arrington's, as we find it better prepared for use and freer of objections than any that we have ever used.

External or local decay of the teeth is that which takes place from external or local causes. We have shown that owing to constitutional and hereditary disease, the teeth may be faulty in structure and are therefore more easily acted upon by external or local agents, but what these agents are and how they decompose so dense a structure as the enamel is not positively understood. Animals living on their natural diet are never subject to caries of the teeth; but domesticate them and feed them on a mixed and hot diet and their teeth become defective. This is illustrated in our western distilleries where they fatten hogs on the refuse which is a kind of hot mush. When once commenced, this must kept up until packed in many instances with Trichina Spiralis for a distant market. For in a short time owing to the hot food and want of use, their teeth become so defective that if turned out into the most luxuriant corn or pea-fields they would die of starvation on account of their not being able to masticate their food.

Again take the peasantry of Scotland, Ireland, Russia, Turkey, and many other countries, where they are, through necessity, compelled to live on a cold and rough diet, and we find their teeth almost free of caries. The negroes on our large plantations before the war were comparatively free of caries, while the cooks and kitchen hands almost in every instance had bad teeth. From the foregoing we think we may safely assert, that the great cause of caries is what is known as chemical action. This is produced by hot food and other agents mixing with a vitiated saliva, forming by fermentation or some other process new agents which are sufficiently corrosive to penetrate the enamel. These new agents, acids, or whatever else they may be, have peculiar ways and places of attack. We do not remember of ever seeing any inferior incisor attacked on the lingual surface, and but in a few cases have we ever seen an inferior molar cuspid or bicuspid decayed on this surface. Now and then we see the superior molars attacked but generally by fissures extending from the posterior coronal surface. The superior incisors are frequently defective on the palatine surface owing mostly to their formation. The most frequent locality of caries is on the coronal surfaces of the molars and bicuspids. The next in order are the approximal surfaces, then the buccal. From this we would argue that owing to the continued brushing of the tongue and the naturally smooth surface of the lingual sides that corrosive agents secure no foothold or place of attack, consequently caries cannot take place. On the coronal surfaces the case is very different. In all the molar and bicuspid teeth there are little pits and fissures where the enamel is thin affording a place for deposit or a kind of minute chemical laboratory, and within these little laboratories the agents of destruction are generated. We have frequently noticed after extracting these teeth with deep pits and fissures, a kind of yellow substance neither mucus or tartar deeply bedded within these places, what this has to do with caries we cannot positively assert, but that it is a generated deposit and that it is of a corrosive character there can be no doubt. This is also generally found on the approximal surfaces whenever they are so situated as to afford lodging for this matter. Again on these surfaces where the teeth are crowded or close together the little abrasion produced in mastication gives a kind of roughness and fine field of action. The buccal surfaces near the margin of the gum are now and then the seat of caries, and wherever such is the case we find some unevenness, or the gum so situated as to assist in lodging the destructive agents. From the foregoing facts we learn then that caries in order to take place must have a foot-hold, some uneven and unmolested surfaces or little pits or fissures from which to operate. This being the case we can safely say whatever will remove this unevenness or the deposits from these pits or rough surfaces will go a great way towards prevention.

As we said before, the lingual and palatine surfaces rarely ever decay because they are smooth, free, and constantly brushed by the tongue. This teaches us that if it were possible to place the other parts of the teeth more liable to decay in a similar condition, we would do much towards prevention of caries. Dr. Arthur of Baltimore has taken a heroic step in this direction. He proposes to file between all the teeth when crowded so as to free their sides of chemical action. This at first may to many sound startling and strange but to the experienced operator it is much more plausible. This filing between means a great deal. It means leaving a nice shoulder, removing every file scratch and burnishing over the filed surface, giving it as smooth and resisting a surface as glass, and at a charge of at least five dollars for every separation. With Dr. Arthur or an experienced operator, we commend this as a safe practice but with the inexperienced or careless operator a very dangerous one. Where teeth are predisposed to decay between in young person's mouths, we are in the habit of extracting one on each side upper and lower. This of course as a general thing should not be done until all the permanent teeth except the dentes-sapientiæ are erupted, or we will have ere long a contracted arch. In the majority of cases where this is necessary, the neighboring teeth on the approximal surfaces are more or less defective and have to be filed and filled, and this filing and filling between others with extracting gives free circulation of the fluids and access to the brush. We think in many cases of this kind we might safely file between all the other unaffected teeth leaving nice polished shoulders, and by thus giving every tooth a little space they would not encroach on each other as is too often the case when one or two spaces are made and the others crowded. Again we would have free surfaces, and the brush with the tongue would keep them so. Approximal decay is by far more difficult to deal with effectually than any other, and we should hail with joy any new theory for preventing its ravages. The tongue in more senses than one teaches us what to do. The thorough use of the brush if timely commenced and persevered in will do for the other surfaces what the tongue does for the lingual and palatine. We find among our patients that there is not one young person in ten who knows how much or often to use the brush, therefore we are in the habit on completing other operations of requesting them to bring their brush If we find this is not what it should be, we instruct what kind to use, and then make them go to work with a watch before their eyes and brush thoroughly for about five minutes, and in many cases if they do not do it energetically we do it for them. This in the presence of a parent gives a lesson that is not easily forgotten, simply requesting young patients to brush their teeth or parents to have it done amounts to nothing, it must be shown or performed so as not to be forgotten.

The idea of wearing so dense a structure as the enamel by brushing, is almost too absurd to be alluded to; and we wonder how Tomes in his work ever advanced such an idea. A few simple experiments on a lathe will convince any one that teeth are never worn by brushing.

When the teeth can be kept perfectly clean without a powder we think it best not to use any. As a general thing the most good derived from powders is the inducement to use the brush for many persons could hardly be induced to use a brush without some kind of powder with a high sounding name, and if by brushing they keep their teeth and gums clean and healthy they are too apt to give the powder the praise when in truth it belongs to the brush.

If the theory of acid generation by chemical decomposition is correct, then it would be reasonable to suppose that he action of this acid on the teeth, to a great extent, might be prevented by an alkaline lotion or dentifrice. The use of this when there is acidity of the saliva we would strongly recommend. A dentifrice composed mostly of precipitated chalk with a little carbonate of soda, or one-fourth of a drachm of carbonate of soda to a glass of water, in brushing, would neutralize the acid. We might add to this the frequent rinsing of the mouth with some such lotion as the above.

As to the true nature of carious matter, we feel obliged to report that comparatively little is known. Microscopy and chemistry have of late been too much engrossed in developing new features in normal tooth structure, to give this subject the attention that its importance demands. Without a correct knowledge of the destructive elements contained in carious dentine, feel that we in its treatment we are like a ship without a compass. Will not some one of our able microscopists and chemists, take this in hand and give us a full and reliable account of the agents at work in this substance? During the last few months we have placed a very fine microscope on our instrument stand, so that we can without the waste of but a few minutes, examine the saliva and to some extent the layers of carious dentine taken from different mouths. This little investigation has sprung many queries, which we cannot positively answer. What is carious dentine? How does it differ from sound tooth structure? Is it the same when detached from as when connected with sound dentine? Will a layer when taken from a tooth and placed under a filling act on the living substance the same as when allowed to remain partially connected with the living? Why are we so particular in the removal of every mite of decay from a cavity before filling? You say because if the minutest mite is left, in the course of time it will increase and surround the filling. Would a mite detached from sound dentine, or any less unobjectionable foreign substance do this? You answer, no. What contagious agent then is there in this mite of decay to do this? How does the same agent produce all these different shades of decay; the black, the white, the yellow, and so on? We are told acetic fermentation, and acids combining with the lime of the tooth form new agents, but what these agents are or how they act on the tooth structure, as they do, microscopy or chemistry, as yet, has not informed us. We would be pleased to answer some of these queries, but as our investigations have not been sufficient to enable us to do so in a positive manner, we think it best to leave the task for some one more competent, believing that it will not be long before we have a better knowledge of the agents contained in carious tooth structure. While we believe in the theory of chemical decomposition by fermentation, yet there are several species of caries, with which we think this has nothing to do. Take for instance, that kind which is called spontaneous abrasion, known by those deep polished groves close to the margin of the gum. These groves are smooth, white, and apparently free of all carious substance, as much so as the surface of the enamel. Yet how rapidly and surely do they wear away unless checked by filling.

In examining the saliva in mouths where we find this class of teeth, we have seen little things which we think are parasites under the microscope. Around the body they look to be about fifty times less than an ordinary sized hair They are large towards the head and of various sizes and shapes, but of the same color. We have not been able to discover them only in certain mouths, and mostly we believe in dyspeptics. The simplest way to see these and innumerable other objects, of which we have no mention, is to take two or three little object glasses, such as are used for examining objects with the microscope, and run the edge of one after the other over the tongue lightly scraping it, thus you have three preparations, giving you in different stages of formation and action all the elements of saliva, and which, when properly examined under the microscope, are little worlds of moving and animated matter. The matter or saliva thus collected from the tongue should be thinly and evenly spread over the surface of the glass to be seen correctly.

In the filling of teeth when the removal of every layer of affected dentine would involve the exposure of the pulp, and where it is thought best to leave a layer, as a capping to the nerve, as this affected dentine is said to contain a corrosive acid, we would recommend the swabbing out of such cavities with a strong solution of carbonate of soda, and then carbolic acid. This treatment we know from experience will be found of great advantage. The alkali neutralizes the acid, and the carbolic acid being a powerful disinfectant destroys the contagious action of other agents. There may seem to some a contradiction here in the so-called carbolic acid, but the trouble is in the name only. Why it is called an acid we do not understand, for it deserves no such term. It is more properly an alkali and its action is more of that nature. We may as well call creosote an acid, for there is little or no difference; one is made from coal, the other from wood; one will crystalize, while the other will not; one is a little stronger in its action than the other, but the effects of both are the same. We might by the same course of reason call molasses an alkali and sugar an acid.

We believe the best protection or capping that can be given the pulp is the one supplied by nature, provided we can neutralize it and allow it to remain with impunity to other parts. This we think can be done, provided we know, as we said before, how to contend with the destructive agents contained therein. On the strength of this belief wherever we suspect the exposure of the nerve, we remove everything around the margin of the cavity, and then reduce the affected part to a state of inactivity. Of course not being positive as to the agents we have to contend with, we are not positive as to the correctness of treatment, all we have to guide us is experience and succees in the course adopted.

We were somewhat influenced towards this course by a criticism from Dr. Flagg on Dr. Arthur's advocating a similar practice. This criticism went on to say as the affected dentine contained a corrosive acid, the practice of using it as a natural capping or protection to the nerve, would not do. Now this to us was instructive, and led us to the above treatment of the dentine, so as to neutralize the acid and other destructive agents.

As to the treatment of pulps we have little to report that was not embraced in our essay on this subject at our last meeting. Considerable attention is now directed to the use of Oxy. Chloride. The action of this on an exposed pulp is peculiar and differs from all other materials. The practice with this material is to flow it over the exposed nerve, and as it hardens, fill and finish. This filling is then allowed to remain several days or weeks, and then about two thirds is removed and replaced by gold. It is contended that in most of cases ossific deposit takes place over the pulp thus treated. We think there are a few cases where such is the case, but in a great majority it does not. In quite a number of cases where we removed these fillings, not through necessity, but merely through curiosity, we found the pulps apparently in a bad condition and felt at a loss to understand how it was possible for them to remain so without trouble. Perhaps this is the most peculiar property of this material excepting that of relieving pain after producing it.

In a few other cases where the teeth were filled similar to the ones just alluded to, and were doing good service, they looked well and gave no trouble, and we found on removing the filling the pulp dried up and apparently no odor. One of the most peculiar cases that has come under our observation was a first sup. molar in the mouth of Dr. Allen, filled by Dr. Ford of Atlanta. This tooth had that peculiar positive shade that indicates devitalization, but on removing the filling we found no sign of pus or odor, although the nerve had shrank away from the floor of the pulp cavity, and to all appearances was gone; on excavating the fangs, about midway we found considerable sensitiveness, similar to that which we meet with in that dryed condition of the pulp where the least irritation gives trouble. What was most peculiar in this case was the devitalized shade of the tooth, indicating absolute disintegration of nerve fibrils and absorption of matter by the tubuli. This tooth, although the pulp was nearly if not quite dead, was not in the condition we expected to find it and perhaps would not occasion trouble. It was well treated apparently, but we were influenced to refill on account of the shade. Much success is claimed in the treatment of pulps by this new material, but sufficient time has not elapsed since its use to justify this claim. A few years will test its merits, and until then, although we are using it and so far can say more in its favor than against it, yet we are unwilling as yet to applaud or condemn.

About 1852, or 1853, Dr. S. P. Hullihen of Wheeling, Va., introduced a new method of treating exposed nerves which he called Risodontrypy, and which a year or two after had many advocates and created considerable ado with old theories. This operation consisted in perforating the nerve midway between the apex and neck by boring a little hole, ordinary needle-size, with a small needle-like drill and spear point. The slightest indication of wounding the nerve without cutting it was the desired result. This slight wound relieved the engorgement by removing a drop or two of blood, causing it to contract from the exposed point and thus allowing filling without pain and generally without trouble afterwards. It was claimed by the advocates of this operation, that ossific deposits took place in the hole made into the fang and also over the exposed nerve. Be this as it may, whether it was owing to the delicacy of the operation or impracticability, it has under its original name, "Risodontrypy," disappeared from practice, but is somewhat revived under another head—we allude to the practice of shaving off a small portion of the pulp when exposed, as introduced by Dr. Allport of Chicago. This of course relieves engorgement of capillary vessels causing the nerve to contract and allowing the filling of the tooth without pain, or much fear of trouble for some time as in risodontrypy. The principle in both is the same, and therefore we cannot claim the latter practice as new. Like Risodontrypy, owing to its delicacy and intricacy, it no doubt in many instances might be successful in the hands of Dr. Allport and a few others, but never in general practice.

Before closing this report, we would call your attention to the great evil that is being inflicted on humanity by the wholesale extracting of teeth as practiced mostly by extracting companies. It is the glory of the men connected with these organizations to keep a record in which every one after leaving some member, is requested to write his name, and some go as far as to form the teeth thus extracted into pyramids for exhibition ; on investigation of some of these pyramids, it has been ascertained that at least two out of every three teeth thus extracted might have been saved. This gives good reason for asserting that nitrous oxide has done more evil than good, for in places where this is used many persons would otherwise think of saving their teeth instead of having them extracted. Again, as the men connected with these organizations, as a general thing, know nothing about what teeth might be saved, they extract with a clear conscience, believing they are doing a service. In country towns and other places where these agents are used the case is not much different, for they are used more to trap in rubber work, than for the little pay they secure for the mere operation of extracting. It is the general custom with many of this class of persons calling themselves dentists, to have a physician present in the use of the anæsthetic, thus acknowledging their inability and want of confidence in the agent used.

Another inducement for this promiscuous extracting is the use of rubber, for persons are informed they can have new teeth inserted for a few dollars, and they in many cases believing a tooth is a tooth, that one is as good as another, do not hesitate to have their teeth removed, to be regretted when too late. But if the evil ended with the extraction, it would not be so bad, but is handed down from parent to child, for like produces like. This holds good with all animal creation, as well as our own species. The physiognomy of the Germans, the French, the English and the Americans all proclaim them as such. If parents have peculiar marked features or teeth, their children generally inherit the same, and if these children intermarry with those having similar marks or defects, these marks or diseases become hereditary; and we may ere long, if this wholesale extracting is persevered in, not only see children without teeth but persons of advanced age that never had any. We have in many places instances of this already, and numerous cases of con tracted and imperfect arches, which can be explained on no other principle. This being the case we must say the various anæsthetics and the use of rubber, have proven a greater curse than a blessing, for they have been the means of sacrificing millions of teeth which we know could have been saved.

